# Reliability of the National Institutes of Health (NIH) Stroke Scale Between Emergency Room and Neurology Physicians for Initial Stroke Severity Scoring

**DOI:** 10.7759/cureus.37595

**Published:** 2023-04-14

**Authors:** Jonathon S Cummock, Kelvin K Wong, John J Volpi, Stephen T Wong

**Affiliations:** 1 Systems Medicine and Bioengineering, Houston Methodist Hospital, Houston, USA; 2 Department of Translational Medical Sciences, Texas A&M University School of Medicine, Bryan, USA; 3 Department of Radiology, Weill Cornell Medicine, Houston, USA; 4 The Ting Tsung and Wei Fong Chao Center for BRAIN, Houston Methodist Hospital, Houston, USA; 5 Department of Radiology, Houston Methodist Academic Institute, Houston, USA; 6 Department of Neurology, Houston Methodist Neurological Institute, Houston, USA; 7 Department of Neuroscience and Experimental Therapeutics, Texas A&M University School of Medicine, Bryan, USA

**Keywords:** stroke, patient transfer, missing data substitution, initial stroke severity scoring, emergency room stroke, stroke assessment, stroke triage, ais (acute ischemic stroke), nihss reliability, nihss (national institutes of health stroke scale)

## Abstract

Introduction: In patients with acute ischemic stroke (AIS), the National Institutes of Health Stroke Scale (NIHSS) is essential to establishing a patient’s initial stroke severity. While previous research has validated NIHSS scoring reliability between neurologists and other clinicians, it has not specifically evaluated NIHSS scoring reliability between emergency room (ER) and neurology physicians within the same clinical scenario and timeframe in a large cohort of patients. This study specifically addresses the key question: does an ER physician’s NIHSS score agree with the neurologist’s NIHSS score in the same patient at the same time in a real-world context?

Methods: Data was retrospectively collected from 1,946 patients being evaluated for AIS at Houston Methodist Hospital from 05/2016 - 04/2018. Triage NIHSS scores assessed by both the ER and neurology providers within one hour of each other under the same clinical context were evaluated for comparison. Ultimately, 129 patients were included in the analysis. All providers in this study were NIHSS rater-certified.

Results: The distribution of the NIHSS score differences (ER score - neurology score) had a mean of -0.46 and a standard deviation of 2.11. The score difference between provider teams ranged ±5 points. The intraclass correlation coefficient (ICC) for the NIHSS scores between the ER and neurology teams was 0.95 (95% CI, 0.93 - 0.97) with an F-test of 42.41 and a p-value of 4.43E-69. Overall reliability was excellent between the ER and neurology teams.

Conclusion: We evaluated triage NIHSS scores performed by ER and neurology providers under matching time and treatment conditions and found excellent interrater reliability. The excellent score agreement has important implications for treatment decision-making during patient handoff and further in stroke modeling, prediction, and clinical trial registries where missing NIHSS scores may be equivalently substituted from either provider team.

## Introduction

In patients with acute ischemic stroke (AIS), the National Institutes of Health Stroke Scale (NIHSS) score is obtained in the initial neurological exam to establish the patient’s initial stroke severity before thrombolytic or thrombectomy treatment. Originally designed for research trials, the NIHSS has been the gold-standard screening tool for AIS clinical research trials for decades [1]. Today, the utility and influence of the NIHSS extend far beyond research trials to its current in-hospital use at the bedside as part of the American Heart Association treatment guidelines for the early management of AIS patients [2], with certifying authorities requiring a baseline NIHSS score before any thrombolytic or thrombectomy intervention and within the first 12 hours for patients not receiving any intervention [3]. While previous research has validated the NIHSS rating system between neurologists, non-neurologist physicians [4], stroke research nurses [5], other groups of clinical staff with varying levels of education/training [6], and differing rater certification environments [7,8], none of these studies specifically evaluated the NIHSS scoring reliability between emergency room (ER) and neurology physicians within the same clinical scenario and timeframe in a large cohort of patients. As the NIHSS score is critical to communication and patient handoff from the ER to the neurology team, the value of a reliable NIHSS score is extremely important for stroke outcomes, as it is strongly correlated with patient discharge, long-term outcomes, and mortality. The goal of this study is to specifically address the key question: does an ER physician’s NIHSS score agree with the neurologist’s NIHSS score in the same patient at the same time in a real-world context? This article aims to critically evaluate the reliability of the NIHSS between ER and neurology teams.

## Materials and methods

The data that supports the findings of this study are available from the corresponding author upon reasonable request, with consideration given to the sensitive clinical nature of the data. All the work presented herein involved a chart review of the patients’ data and was approved by the Houston Methodist Research Institute Institutional Review Board with an informed consent waiver (approval: IRB0607-0094 NeuroCADD).

Patient selection

Data were retrospectively collected from a registry of 1,946 patients being evaluated for AIS at Houston Methodist Hospital (a comprehensive stroke center) from 05/2016 - 04/2018 [9]. To be included for analysis, the patient had to have two NIHSS scores, one from the neurology team and the other from the ER provider, both performed within the same clinical scenario (pre-treatment, post-treatment, or no treatment) and timeframe (<1 hour of time difference). We define treatment as an intervention with thrombolysis, thrombectomy, or both, and no treatment as medical management only. To mitigate the effect of the time delay, we manually reviewed the electronic medical records of patients with any difference between the ER and neurology NIHSS scores to ensure that no clinical change, such as hemorrhagic transformation, had occurred over the <1 hour lapsed timeframe. We also assessed whether the documentation differed for other reasons, such as errors or omissions. Consequently, 10 cases were excluded due to incorrect documentation of the NIHSS score (e.g., positive stroke findings recorded by the physician that were either absent or incorrectly scored in the documented NIHSS score). Ultimately, 129 patients were included in the analysis. All providers in this study were NIHSS rater-certified.

Evaluation metrics and statistical analysis

Analysis was performed using R software version 4.2.2 (R Foundation for Statistical Computing, Vienna, Austria). Rater reliability was evaluated using intraclass correlation coefficient (ICC) statistics following the Shrout and Fleiss convention [10]. ICC values were calculated with a 95% confidence interval using the R psych package version 2.2.5 [11]. ICC2 was selected as the most applicable reliability statistic, calculated by a single-rating, absolute-agreement, two-way random-effects model with two raters across the 129 subjects. Calculated ICC values closer to 1 indicate better reliability, with an ICC value of 1 representing perfect reliability. ICC values less than 0.5, between 0.5 and 0.75, between 0.75 and 0.9, and greater than 0.90 are indicative of poor, moderate, good, and excellent reliability, respectively [12].

## Results

**Figure 1:**
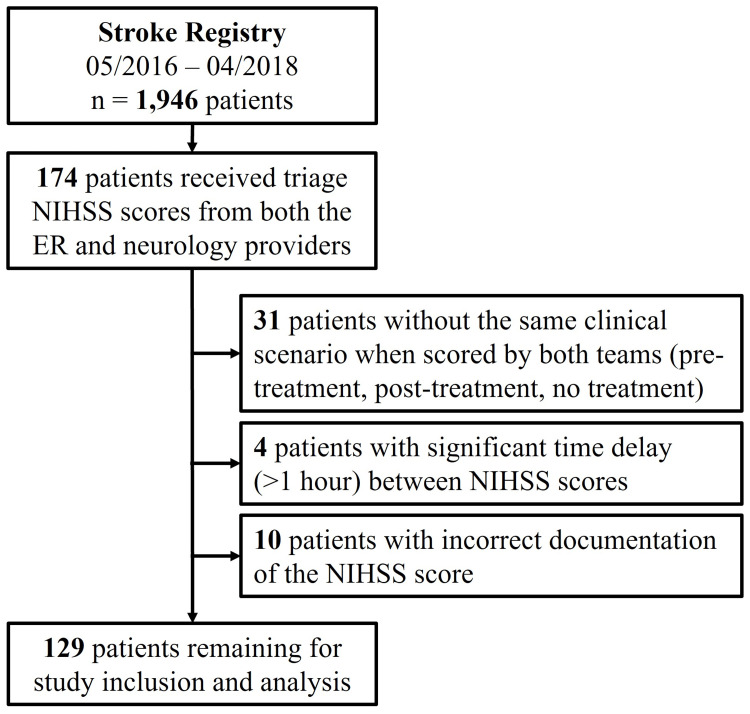
Study inclusion flow diagram Inclusion and exclusion criteria for patients from a stroke registry evaluated for acute ischemic stroke at Houston Methodist Hospital from 05/2016 to 04/2018.

We evaluated the triage NIHSS scores of 129 patients treated at a comprehensive stroke center. Patient inclusion criteria are shown in Figure 1. The patients’ mean age was 65.3 years old (SD = 15.6), with a range of 23 to 94 years old, and 62 (48.1%) were men. In this patient group, stroke vascular territory was evenly distributed between the left (32.56%) and right (31.01%) hemispheres. The patients’ clinical characteristics are summarized in Table 1. Patients were treated according to eligibility criteria, following current guidelines. The majority of patients received medical management only (58.91%), while the remainder received interventional treatment, including tPA (23.26%), mechanical thrombectomy (9.30%), or both (8.53%).

**Table 1:** Patient characteristics Characteristics of the 129 patients included for analysis are shown.

Patient characteristics	Overall, n = 129
Age, years (SD)	65.26 (15.61)
Sex (Men), no. (%)	62 (48.06)
NIHSS score from ER, no. (SD)	6.43 (6.92)
NIHSS score from Neurology, no. (SD)	6.88 (7.01)
Modified Rankin Scale (mRS) score, no. (SD)	1.95 (1.84)
Stroke vascular territory	
Left anterior cerebral artery, no. (%)	5 (3.88)
Left lenticulostriate artery, no. (%)	4 (3.10)
Left middle cerebral artery, no. (%)	15 (11.63)
Left posterior cerebral artery, no. (%)	11 (8.53)
Left watershed territory, no. (%)	7 (5.43)
Right anterior cerebral artery, no. (%)	3 (2.33)
Right lenticulostriate artery, no. (%)	9 (6.98)
Right middle cerebral artery, no. (%)	13 (10.08)
Right posterior cerebral artery, no. (%)	7 (5.43)
Right watershed territory, no. (%)	8 (6.20)
Unknown, no. (%)	47 (36.43)
Treatment	
Medical management only, no. (%)	76 (58.91)
Tissue plasminogen activator (tPA), no. (%)	30 (23.26)
Mechanical thrombectomy (MT), no. (%)	12 (9.30)
tPA with MT, no. (%)	11 (8.53)

**Figure 2:**
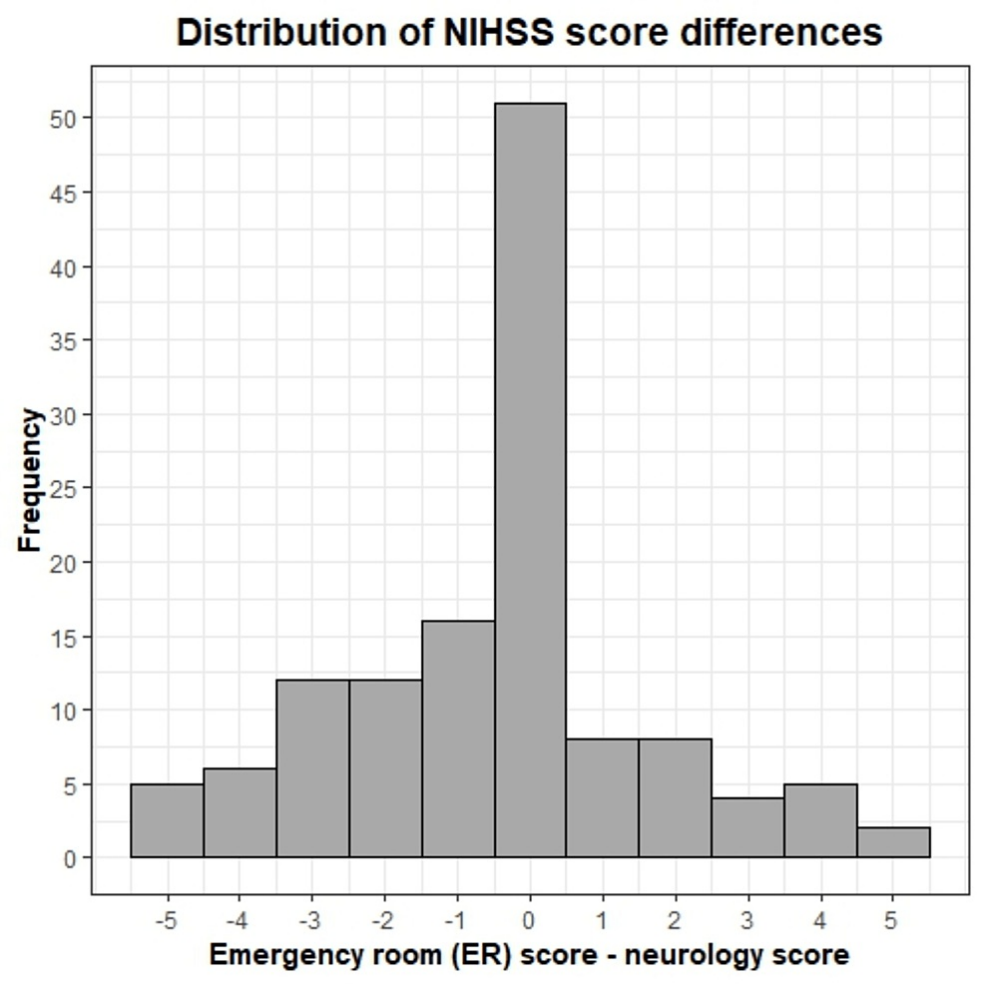
NIHSS score differences between provider groups Histogram of the distribution of NIHSS score differences calculated from the emergency room (ER) score – the neurology score. The data is centered around the mean of -0.46 with a standard deviation of 2.11.

A histogram of the distribution of the NIHSS score differences (ER score - neurology score) is shown in Figure 2, with a mean difference of -0.46 and a standard deviation of 2.11. The range of score differences between the provider teams was ±5 points. NIHSS scores from the ER team (mean = 6.43; SD = 6.92) and neurology team (mean = 6.88; SD = 7.01) were compared with a two-sided Welch’s paired t-test and found to be significantly different (p < 0.05). Further analysis among treatment groups showed that NIHSS scores between the ER and neurology teams differed significantly in patients who received any treatment (tPA, MT, or both; p < 0.05) but not in patients who received medical management only (p = 0.18). The patient subgroup comparison results are summarized in Table 2.

**Table 2:** NIHSS score comparison in patient subgroups NIHSS scores from the emergency room (ER) and neurology teams were evaluated in a subgroup analysis by triage stroke severity and by treatment category. NIHSS scores by team were compared with a two-sided Welch’s paired t-test.

NIHSS scores from the ER and neurology teams compared	t-statistic	Degrees of Freedom	P-value	Mean difference (95% CI)
NIHSS scores overall	-2.46	128	0.02	-0.46 (-0.83 – -0.09)
Low severity (scores <6)	-0.31	73	0.76	-0.07 (-0.50 – 0.37)
Moderate severity (scores 6 – 18)	-2.76	41	0.01	-1.00 (-1.73 – -0.27)
High severity (scores >18)	-1.48	12	0.17	-0.92 (-2.28 – 0.44)
Grouped by treatment				
Medical management only	-1.35	75	0.18	-0.32 (-0.78 – 0.15)
Tissue plasminogen activator (tPA)	-2.29	29	0.03	-0.90 (-1.70 – -0.10)
Mechanical thrombectomy (MT)	-1.10	11	0.29	-0.83 (-2.50 – 0.83)
tPA with MT	0.32	10	0.76	0.18 (-1.09 – 1.45)

ICC values were calculated to determine the level of scoring agreement between the two provider teams. The ICC kappa value was 0.95 (95% CI, 0.93 - 0.97), indicating excellent reliability of NIHSS scoring between the ER and neurology teams. The F-test score was 42.41, with a p-value of 4.43E-69, further supporting the excellent agreement between the two provider teams. The full ICC results are shown in Table 3.

**Table 3:** Intraclass correlation between emergency room and neurology physicians The full results of intraclass correlation (ICC) are shown. ICC2 was selected as the most applicable reliability statistic, calculated by a single-rating, absolute-agreement, two-way random-effects model with two raters. Calculated ICC values closer to 1 indicate better reliability, with an ICC value of 1 representing perfect reliability. ICC values less than 0.5, between 0.5 and 0.75, between 0.75 and 0.9, and greater than 0.90 are indicative of poor, moderate, good, and excellent reliability, respectively.

Intraclass correlation coefficients	Type	ICC (95% CI)	F-test	Degrees of Freedom 1	Degrees of Freedom 2	P-value
Single raters absolute	ICC1	0.95 (0.93 – 0.97)	40.81	128	129	1.61E-68
Single random raters	ICC2	0.95 (0.93 – 0.97)	42.41	128	128	4.43E-69
Single fixed raters	ICC3	0.95 (0.94 – 0.97)	42.41	128	128	4.43E-69
Average raters absolute	ICC1k	0.98 (0.97 – 0.98)	40.81	128	129	1.61E-68
Average random raters	ICC2k	0.98 (0.96 – 0.98)	42.41	128	128	4.43E-69
Average fixed raters	ICC3k	0.98 (0.97 – 0.98)	42.41	128	128	4.43E-69

## Discussion

In patients with AIS, the NIHSS score is a critical component for establishing stroke triage severity and has a direct impact on guiding treatment decisions. The results of this study are consistent with previous research that has demonstrated the reliability of the NIHSS score for stroke severity assessment. However, this study is unique in that it specifically evaluated the reliability of the NIHSS score obtained by both ER and neurology physicians in a real-world clinical setting. We compared NIHSS scores from 129 patients evaluated by both teams under matching time and clinical conditions and observed overall excellent inter-rater reliability. Given that all the providers in our study were NIHSS rater-certified, these results have important implications for patient care and outcomes. It ensures that accurate and consistent information is being communicated between providers, which is critical for effective patient handoff and treatment decisions. Additionally, the strong agreement between the provider teams supports the substitution of the triage NIHSS score from the ER team in studies or clinical trial registries where the score may be missing from the neurology team. This has important applications for retrospective studies and post-hoc analyses where the data has already been collected.

Recent efforts have been made to extend the use of the NIHSS beyond research and in-hospital use to prehospital stroke triage. A Norwegian study of prehospital emergency medical services compared NIHSS scores from rater-certified paramedics to stroke physicians in 274 patients and found moderate agreement (kappa of 0.58) between the groups [13]. As prehospital use of the NIHSS rises among emergency responders, our study validating the interrater reliability of triage NIHSS scores between ER and stroke providers (kappa of 0.95) takes on increased relevance as a patient’s care transitions from EMTs or paramedics to the ER or stroke providers, depending on individual hospital resources and practices.

Our study has several limitations. Of the 1,946 patients in the stroke registry, we initially identified 174 patients who had received triage NIHSS scores from both the ER and neurology providers. As the patients in this study were evaluated at a comprehensive stroke center, in the majority of cases, the neurology team was the initial team to evaluate the patient with NIHSS scoring. In these cases, typically only the neurology NIHSS scores were documented, which narrowed the study group. Furthermore, we excluded 10 cases due to erroneous physician documentation of the NIHSS score. These score discrepancies were most often caused by positive stroke clinical exam findings documented elsewhere in the electronic medical record but not reflected in the NIHSS score. For example, one provider documented “Right arm flaccid” in the objective findings free text portion of their clinical note, but the NIHSS documentation for right arm motor drift was scored as zero, indicating no weakness. We found that these errors were not necessarily the result of inadequate examination but rather poor documentation. Additionally, we were not able to perform a subset analysis of individual test item reliability as not all the patients had individual NIHSS test item scores recorded. Since hospital scales and scoring systems can be used to derive clinically significant information, this highlights the importance of complete and proper documentation by physicians in the electronic medical record. This study was conducted at a single comprehensive stroke center in which all the providers were NIHSS rater certified. Further investigation with additional patients from multiple sites, including primary and comprehensive stroke centers, is warranted to confirm these results.

## Conclusions

We evaluated triage NIHSS scores performed by ER and neurology providers under matching time and treatment conditions and found excellent interrater reliability. The excellent overall agreement in NIHSS scores between ER and neurology providers has important implications for treatment decisions and patient handoff. This result is also relevant to stroke disease modeling, prediction, and clinical trial registries where missing NIHSS scores may be equivalently substituted from either provider team.
